# A Comparison of a Fully Covered and an Uncovered Segmented Biodegradable Esophageal Stent in a Porcine Model: Preclinical Evaluation of Degradation, Complications, and Tissue Reactions

**DOI:** 10.1155/2016/8690858

**Published:** 2016-02-28

**Authors:** Yadong Feng, Chunhua Jiao, Yang Cao, Ye Zhao, Yanfang Chen, Lin Fang, Ruihua Shi

**Affiliations:** ^1^Gastroenterology Department, The First Affiliated Hospital of Nanjing Medical University, 300 Guangzhou Road, Nanjing 210029, China; ^2^Gastroenterology Department, Zhongda Hospital, Southeast University, 87 Dingjiaqiao, Nanjing 210009, China

## Abstract

*Aims*. This study was to compare the degradation, complications, and tissue reactions of two segmented biodegradable esophageal stents in a porcine model.* Methods*. Uncovered biodegradable segmented stents and fully covered biodegradable segmented stents (FCBDS) were transplanted into the porcine esophagus lumen. Data on biodegradation, complications, and tissue reactions were collected and compared.* Results*. All animals kept good general conditions. No severe complications and stents migration occurred. Stents degradation commenced at week 3. Compared with uncovered stents, stents structure breakage and complete stents absorption in FCBDS were postponed for 1-2 weeks. Hyperplasia was prominent at early stage and ameliorated at late stage after stents insertion. Tissue reactions in FCBDS were milder than those in uncovered stents in the early stage. A longer degradation period was present in FCBDS than in uncovered stents, while FCBDS induced tissue reaction at late stage was mild.* Conclusions*. Biodegradable esophageal stents with a segmented trunk may be further evaluated in refractory benign esophagus strictures. This FCBDS may be advantageous compared with uncovered stents for a longer degradation period.

## 1. Introduction

Refractory benign esophagus strictures (RBESs) are commonly encountered problems to gastroenterologists. The aims of treatments of RBESs are to relieve symptoms of dysphagia, to avoid complications, and to prevent recurrence [1]. Endoscopic treatment options such as dilation, stent placement, and needle knife incision have been established for RBESs [1–4]. Endoscopic stent placement is proposed as the most well-accepted and effective method [5], due to its favorable outcomes for longer lasting dilation effects, ability of keeping luminal patency, simultaneous stretching of the strictures, and low incidence of morbidity and mortality [4, 5]. Self-expanding metal stents (SEMSs) and fully covered metal stents (FCSEMSs) were with high risk of complications, such as perforation, new stricture formation, and ulceration [6–8]. Self-expandable plastic stents (SEPSs) are approved by FDA for RBESs but are with high incidence of migration for a full covering design [9]. Biodegradable esophagus stent is a substitute of SEPSs. Among biodegradable materials, polydioxanone is mostly used for biodegradable stents for good histocompatibility [10]. A biodegradable esophageal stent made of polydioxanone, ELLA-BD (Hradec-Kralove, Czech Republic), is commercially available from 2008. Data on long-term clinical successful rate of ELLA-BD stent is from 26% to 60% in limited samples in humans [1, 4]. Furthermore, a biodegradable stent is not soft and flexible. Preclinical studies of biodegradation procedures and complications of biodegradable stents in animal models are lacking.

We introduced two new polydioxanone esophageal stents; both were with a segmented trunk. A fully covered design of poly-L-lactic acid membrane was introduced to achieve a fully covered biodegradable stent (FCBDS). These two esophageal stents are softer and more flexible than a whole knitted one and may be more suitable for RBESs. This study is to evaluate biodegradation, complications, and tissue reactions of these two stents in a porcine model.

## 2. Materials and Methods

### 2.1. Study Design

All twelve Bama pigs with weight 33–36 kg experienced a 2-week mandated quarantine period and were divided into two groups, which received uncovered biodegradable stents (*n* = 6, group A) and FCBDS (*n* = 6, group B) insertion. All animals received human care in accordance with the Principle of Laboratory Animal Care and the Guide for the Care and Use of Laboratory Animals. All procedures were approved by the Ethics Committee of Nanjing Medical University, Nanjing, China, and GATEWAY Medicine Innovation Center, Shanghai, China. General conditions were observed once per day. Stent biodegradation, complications, and tissue reactions were evaluated once a week. End points were as follows: (1) animals were moribund; (2) all stents were fully degraded and normal esophageal mucosa was present. Euthanasia was executed at end points at GATEWAY Medicine Innovation Center.

### 2.2. Stents and Delivery System

**Figure 1:**
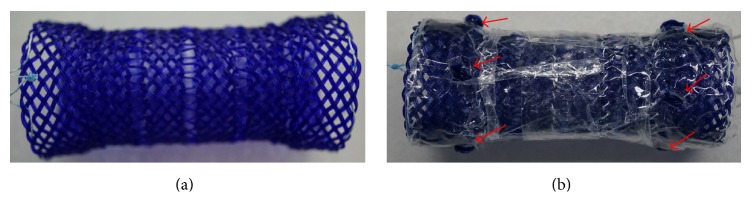
Two segmented polydioxanone made biodegradable esophageal stents. (a) An uncovered stent. The stent was with a segmented 28 × 80 mm trunk and two 30 × 10 mm cup ends. The trunk was composed of four independent segments, connected with 4 mm long polydioxanone monofilament. Each segment was woven by double polydioxanone filaments and was 10 mm in length. (b) A fully covered biodegradable stent. A layer of poly-L-lactic acid membrane, 0.5 mm in thickness, was covered to an uncovered stent. Four protrusions, 3 mm in diameter, were attached to each cup end (red arrows).

All stents were for the present animal study only. All biodegradable esophageal stents, with a segmented 28 × 80 mm trunk and two 30 × 10 mm cup ends, were manufactured from polydioxanone fibers by a nonvascular stent manufacturer (the Micro-Tech Company, Nanjing, China). The stent trunk was composed of four independent segments, connected with 4 mm long polydioxanone monofilament. Each segment was woven by double polydioxanone filaments and was 10 mm in length. In FCBDS, a layer of poly-L-lactic acid membrane, 0.5 mm in thickness, was covered to an uncovered stent. Four protrusions, 3 mm in diameter, were attached to each cup end. A local compression test was performed using a radial force measurement machine (designed by Southeast University, Nanjing, China) and a force gauge (Instron, UK) in an oven at 37°C. The radial resistance force was 18.93 ± 4.16 N and the chronic outward force was 10.21 ± 2.82 N at 14 mm expansion. These two stents were shown in Figure 1.

**Figure 2:**
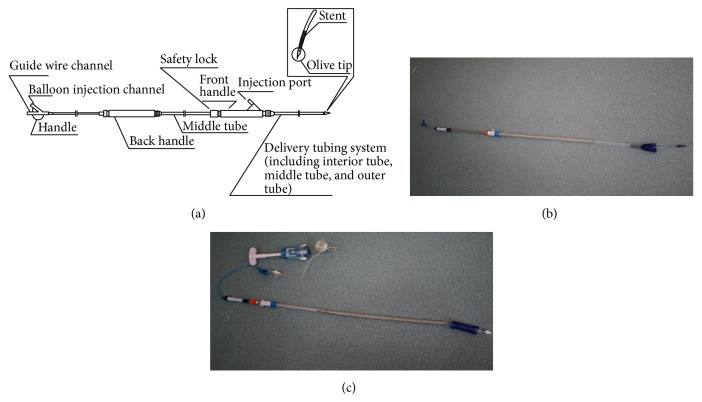
The stent delivery system. (a) The schematic diagram of the stent delivery system. The delivery system consists of three coaxial tubes. The outer tube serves to constrain the stent until being retracted during the stent deployment. The interior tube contains a central lumen that accommodates a 0.035 in./0.89 mm guide wire. In addition, the delivery system has a balloon catheter. (b) A stent was partly accommodated into a delivery system. The outer tube was 22 Fr. A 0.035-inch guide wire was inserted into the interior tube. (c) The balloon catheter was designed at 28 × 100 mm size; the balloon and a pump for balloon dilation were showed.

The delivery system consisted of three coaxial tubes, including an interior tube, a middle tube, and an outer tube (Figure 2). The outer tube was 22 Fr and served to constrain the stent being retracted. The interior tube contained a central lumen that accommodates a 0.035 inch/0.89 mm guide wire. The delivery system had a 28 × 100 mm balloon catheter which could help the stent to be dilated.

### 2.3. Stent Deployment and Follow-Up Gastroscope Examinations

All procedures were performed under general anesthesia induced by an intravenous injection of fentanyl (0.01 mg·kg^−1^·h^−1^) and propofol (8 mg·kg^−1^·h^−1^). A gastroscope was introduced and a 0.035-inch guide wire was inserted into the stomach. The stent was manually loaded into the delivery system and introduced into the esophageal lumen. The stent was deployed at the middle part of the esophagus. Immediate endoscopy was performed to ensure the position of the stent. Balloon dilation with 1 atm for 1 min was performed. The delivery system was withdrawn after stent placement. Follow-up endoscopy was conducted weekly until stents were fully degraded and mucosa turned normal. Stent location, migration, degradation, and complications were recorded. All endoscopy procedures were performed by Dr. Yadong Feng, Dr. Chunhua Jiao, and Dr. Yang Cao.

### 2.4. Definition of Complications and Tissue Reactions

In-procedure complications included perforation and mucosal bleeding. Postprocedure complications included mucosa hemorrhage, esophageal ulcer, stent migration greater than 2 cm, and hyperplasia. A 6-grade scoring system for tissue reaction (TRS, Table 1) was proposed for evaluation of the severity of esophagus tissue lesions, according to tissue inflammation, size of tissue nodules, and patency of esophagus lumen. This system was modified according to two published tracheal tissue reactions scoring systems [10, 11]. All TRS scores were determined by Dr. Yadong Feng, Dr. Chunhua Jiao, Dr. Yang Cao, and Dr. Ruihua Shi.

### 2.5. Statistical Analysis

Data were analyzed in IBM SSPS 19.0. The Mann-Whitney *U* test and ANOVA analysis were used to assess the statistical significance of the differences, with *P* values less than 0.05 considered statistically significant.

## 3. Results

The time of preparation of this delivery system was 5.25 ± 1.03 min. Since ELIA biodegradable stent is not available in China, a comparison between these two segmented stents and ELIA stent was not performed. Technical success rate of stents insertion was 100%. No death, decreased food intake, abnormal behavior, weight loss, and malnutrition of animals were found. No in-procedure complications occurred. No perforation, ulcer, hemorrhage, and migration more than 2 cm occurred. All stents degraded at week 11 and normal esophageal mucosa was present at week 12. Euthanasia was performed at week 12.

### 3.1. Comparison of Stent Biodegradation

**Figure 3:**
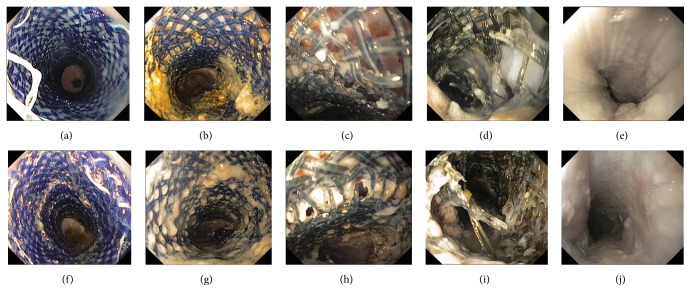
Endoscopic view of degradation procedures of an uncovered biodegradable stent ((a)–(e)) and a fully covered biodegradable stent ((f)–(j)) in vivo. (a) Week 0: immediately after an uncovered stent transplantation. (b) Week 1 after stent transplantation: no sign of degradation was present. (c) Week 3: larger stent meshes and partial separation of the stents from the esophageal wall at the stent trunk were present. (d) Week 8: stent structure breakage occurred. (e) Week 9: complete stent degradation of an uncovered biodegradable stent. (f) Week 0: immediately after a fully covered stent transplantation. (g) Week 1 after stent transplantation: no sign of degradation was present. (h) Week 3: larger stent meshes, partial separation of the stents from the esophageal wall at the stent trunk, and poly-L-lactic acid membrane fracture were present. (i) Week 9: stent structure breakage occurred in a fully covered biodegradable stent. (j) Week 10: complete stent degradation of a fully uncovered biodegradable stent.

The endoscopic biodegradation procedures were showed in Figure 3. In groups A and B, all stents degradation commenced at week 3. Signs of larger meshes and partial separation of the stents from the esophageal wall, especially at the stent trunk, were present. In group B, poly-L-lactic acid membrane fracture was present since week 3. From week 4 to week 6, polydioxanone fibers became thinner and the stents were discolored with larger meshes. Stent structures breakage was present at weeks 7-8 in group A and weeks 8-9 in group B (7.17 ± 0.41* versus *8.16 ± 0.41, *P* = 0.002). In group B, fragmentized poly-L-lactic acid membrane could be observed when stents were fractured at weeks 8-9. Complete stent disintegration occurred at weeks 9-10 in group A and at weeks 10-11 in group B (9.16 ± 0.40* versus *10.50 ± 0.55, *P* = 0.001).

### 3.2. Tissue Reaction Evaluations

**Figure 4:**
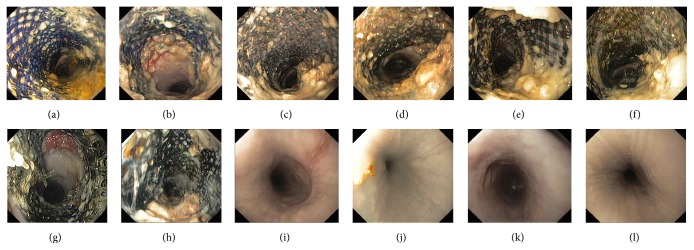
Endoscopic view of tissue reactions caused by an uncovered biodegradable stent. ((a) and (b)) Weeks 1 and 2: circumferential inflammation and nodular hyperplasia ingrowth. ((c)–(f)) Weeks 3–6: significant tissue reactions at contact points between the stents and the esophageal mucosa, with confluent nodules. ((g)-(h)) Weeks 7-8: confined tissue reactions, inflammation, and confluent nodular tissues gradually became alleviated. (i) Week 9: light scars were left. ((j)–(l)) Weeks 10–12: normal esophageal mucosa was present. TRS scores for weeks 1–12 were 2, 2, 3, 3, 3, 3, 2, 2, 0, 0, 0, and 0 for this case.

**Figure 5:**
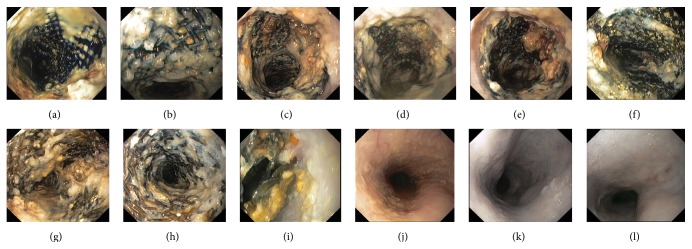
Endoscopic view of tissue reactions caused by a fully covered biodegradable stent. ((a) and (b)) Weeks 1 and 2: circumferential inflammation and nodular hyperplasia. ((c)–(e)) Weeks 3–5: significant tissue reactions at contact points between the stents and the esophageal mucosa, with confluent nodules. ((f)–(j)) Weeks 6–10: confined tissue reactions, inflammation, and confluent nodular tissues. (k) and (l) Weeks 11-12: normal esophagus mucosa. TRS scores for weeks 1–12 were 2, 2, 3, 3, 3, 2, 2, 1, 1, 1, 0, and 0 for this case.

Tissue reactions were diminished at the late stage. Circumferential mucosal inflammation and nodular hyperplasia ingrowth were present at weeks 1-2. Hyperplastic tissues protruded into the esophageal lumen through the stent mesh. At week 3, tissue reactions were found at contact points between the stents and the esophageal mucosa, with confluent nodules at 5–10 mm diameter. Mucosal tissue reactions were confined from week 4. Hyperplasia was prominent at the contact sites between the stents and the esophageal wall. Inflammation decreased and confluent nodular tissues gradually became smaller. When the stents were fractured and completely decomposed, light scars were left. The normal esophagus mucosa was present 1-2 weeks after stents degradation. Tissue reactions duration in group B was longer than it in group A. Compared with group A, degree of tissue reactions in group B was decreased at weeks 1–3 and was more severe after week 9. Although TRS scores of group B at weeks 4–8 were higher than those of group A, there was no statistical significance. In group B, stent induced hyperplasia was mild at the stage and rapidly disappeared after complete stents degradation. Tissue reactions caused by an uncovered biodegradable stent and FCBDS were showed in Figures 4 and 5. A semiquantitative analysis of TRS sores for groups A and B was listed in Table 2.

## 4. Discussion

There are two available biodegradable stents, the first one is polydioxanone made ELLA-BD stent, and the second one is poly-L-lactic acid- (PLLA-) BD stent (Marui Textile Machinery, Osaka, Japan). Due to a tendency of early stent migration [11–13], PLLA-BD stent was not further evaluated for its applications. The ELLA-BD stent was with a migration at 20% and a success rate at 26–60%. However, ELLA-BD stent may induce significant hyperplastic tissue reactions [14, 15]. Up to date, the major limitations of biodegradable stent in RBESs are hyperplastic tissue reactions and stent migration [1, 4]. In this study, uncovered and fully covered polydioxanone biodegradable esophageal stents with a segment trunk were introduced. To decrease risks of tissue hyperplasia and migration, the full covered stent was designed by covering a layer of poly-L-lactic acid membrane and attaching small protrusions. Compared with wholly knitted stents, these two segmented stents were softer and more flexible and nontraumatic and might be more suitable for long, angulated, and irregular RBESs. Bama pig, a miniature swine, was selected as the model for similar esophageal pH value to that of human. To fit Bama pigs' esophageal lumen, all stents were in a larger diameter.

In stent deployment procedures, immediate balloon dilation after stents deployment was technically required due to insufficient stent expansion. This is in accordance with the study in human reported by Canena et al. [16]. No severe complications as perforation, hemorrhage, fistula, and stent migration happened. All animals kept good conditions in whole procedures. These suggested all stents were with good safety.

Stent biodegradation of two stents was evaluated and analyzed. Our data on stent biodegradation are slightly earlier than results from ELLA-BD stent that ELLA-BD stent degradation started at 4-5 weeks and completely finished within 2-3 months in vivo [1, 4]. This may due to a slighter acid pH [17] in Bama pigs esophagus, which was 6.5–6.8 and may accelerate biodegradation procedure. In our study, the degradation duration of covered stent in group B was longer than that in group A. Although stents degradation in two groups starts at week 3, stent structure breakage and complete stent absorption in group B were postponed for 1-2 weeks. This may be correlated with the full covering design of poly-L-lactic acid membrane. The poly-L-lactic acid biodegradation period in vivo is 3–6 months [4], which is longer than that of polydioxanone. The delayed bioabsorption of poly-L-lactic acid membrane may protect polydioxanone fibers from digestive juice, diminish the effect of hydrolysis, and retard the degradation period of polydioxanone. In complex RBESs, multiple restenting is always needed to achieve a long dilation period and to keep patients symptom-free [1]. A prolonged degradation period of a biodegradable stent in treatment of complex RBESs may be helpful by avoiding repeated endoscopy. There were two reasons for less frequent stent migration. First, implementation of a large balloon dilation after stent deployment may reduce risks of early stent migration [16]. Second, the biodegradable material induced hyperplasia may be helpful to fixing stents in position [18].

According to available data, the development of polydioxanone stent induced tissue hyperplasia was self-limiting and reversible in the airway and the urine model [10, 19, 20]. Tissue reactions of these two stents were obvious in the three weeks and then ameliorated. Despite hyperplasia in the early stage in groups A and B, no obvious tissue ingrowth was observed and esophageal lumen patency was present. According to our results, tissue reactions were prominent at the early stage and then ameliorated at the late stage after stents transplantation. This is different with the data from ELLA-CS BD stent [14, 15, 18] that serious tissue hyperplasia happened at late stage and caused recurrent dysphagia. Our results showed these segmented stents may avoid risks of late stage hyperplasia. Tissue reactions in two groups were compared. According to TRS score, tissue reactions at the early stage in group B were milder than in those uncovered stents in group A for the full covering design [7, 21, 22]. When signs of stents biodegradation were present, there was no difference of TRS between two groups at weeks 4–8. However, due to a longer degradation period, tissue reactions in group B remained a longer duration than those in group B. Though FCBDS induced tissue reactions even at week 8, the degree of hyperplasia was mild and acceptable with low TRS scores [10, 11]. Thus we argue that FCBDS may be more advantageous than the uncovered stent for causing milder early tissue reactions.

This study was designed to evaluate the biodegradation and safety of two segmented biodegradable esophageal stents in a pig model. Both stents were with novel biodegradation processes, self-limited tissue reactions, and low risks of late stage hyperplasia in vivo. The full covered stents may be more advantageous for causing milder tissue reactions in the early stage. These two segmented biodegradable esophagus stents may be promising for further evaluations and the full covering design with attached protrusions may be more advantageous. Stents at 14, 16, 18, 20, and 22 mm diameter will be applied for further clinical evaluations. We argue further trials to evaluate these segmented biodegradable stents in RBESs instead of whole knitted stents.

## Figures and Tables

**Table 1:** Grading system for degree of esophageal tissue reaction: TRS.

Grade	Criteria and definitions
0	Normal tissue without inflammation
1	Confined inflammation; hyperplastic tissues at proximal, distal part or meshes with nodes not exceeding stent interior plane
2	Hyperplastic tissue ingrowth protruding from the stent interior plane with a single nodule not larger than 5 mm in diameter
3	Hyperplastic tissue ingrowth protruding from the stent interior plane with a nodule not larger than 10 mm in diameter
4	Diffuse inflammation; hyperplastic tissue ingrowth protruding from the stent interior plane with a node larger than 10 mm in diameter
5	Esophageal lumen blockage caused by tissue ingrowth or overgrowth

**Table 2:** TRS for groups A and B from week 1 to week 12.

	TRS score		*P* value
	Group A	Group B	
W1	2.50 ± 0.55	1.67 ± 0.52	0.022
W2	3.33 ± 0.52	2.50 ± 0.54	0.022
W3	3.67 ± 0.52	2.52 ± 0.50	0.04
W4	2.50 ± 0.55	2.33 ± 0.52	0.599
W5	1.83 ± 0.41	2.33 ± 0.52	0.092
W6	1.50 ± 0.55	2.17 ± 0.75	0.110
W7	1.56 ± 0.75	1.83 ± 0.75	0.664
W8	1.00 ± 0.00	1.33 ± 0.52	0.145
W9	1.00 ± 0.00	1.66 ± 0.52	0.01
W10	0.00 ± 0.00	1.00 ± 0.61	0.003
W11	0.00 ± 0.00	0.83 ± 0.41	0.001
W12	0.00 ± 0.00	0.00 ± 0.00	/

TRS scores were expressed as mean ± SD. Compared with group A, degrees of tissue reactions in group B were milder at weeks 1–3, with no significance at weeks 4–8, and more severe at weeks 9–11.
